# Gomafu lncRNA knockout mice exhibit mild hyperactivity with enhanced responsiveness to the psychostimulant methamphetamine

**DOI:** 10.1038/srep27204

**Published:** 2016-06-02

**Authors:** Joanna Y. Ip, Masamitsu Sone, Chieko Nashiki, Qun Pan, Kiyoyuki Kitaichi, Kaori Yanaka, Takaya Abe, Keizo Takao, Tsuyoshi Miyakawa, Benjamin J. Blencowe, Shinichi Nakagawa

**Affiliations:** 1 RNA Biology Laboratory, RIKEN, 2-1 Hirosawa, Wako 351-0198, Japan; 2 Banting and Best Department of Medical Research, Donnelly Centre, University of Toronto, Toronto, Ontario M5S 3E1, Canada; 3 Laboratory of Pharmaceutics, Department of Biomedical Pharmaceutics, Gifu Pharmaceutical University, 1-25-4 Daigakunishi, Gifu 501-1196, Japan; 4 Laboratories of Animal Resource Development and Genetic Engineering, RIKEN Center for Life Science Technologies, 2-2-3 Minatojima Minami, Chuou-ku, Kobe 650-0047, Japan; 5 Section of Behavior Patterns, Center for Genetic Analysis of Behavior, National Institute for Physiological Sciences, Okazaki, Japan; 6 Division of Animal Resources and Development, Life Science Research Center, University of Toyama, 2630 Sugitani, Toyama, 930-0194, Japan; 7 Division of Systems Medical Science, Institute for Comprehensive Medical Science, Fujita Health University, Toyoake, Japan; 8 Department of Molecular Genetics, University of Toronto, Toronto, Ontario M5S 1A8, Canada

## Abstract

The long noncoding RNA Gomafu/MIAT/Rncr2 is thought to function in retinal cell specification, stem cell differentiation and the control of alternative splicing. To further investigate physiological functions of Gomafu, we created mouse knockout (KO) model that completely lacks the Gomafu gene. The KO mice did not exhibit any developmental deficits. However, behavioral tests revealed that the KO mice are hyperactive. This hyperactive behavior was enhanced when the KO mice were treated with the psychostimulant methamphetamine, which was associated with an increase in dopamine release in the nucleus accumbens. RNA sequencing analyses identified a small number of genes affected by the deficiency of Gomafu, a subset of which are known to have important neurobiological functions. These observations suggest that Gomafu modifies mouse behavior thorough a mild modulation of gene expression and/or alternative splicing of target genes.

Recent studies using high-throughput RNA-sequencing (RNA-seq) technologies have revealed that mammalian genomes produce a large number of functional non-protein-coding RNAs (ncRNAs) that are involved in different cellular processes^1^,^2^. Among these ncRNAs, those that are longer than 200 bp and possess no protein-coding capacity are arbitrarily grouped as long non-coding RNAs (lncRNAs). The most well-studied function of lncRNAs is to act as epigenetic regulators and transcription regulators through binding to proteins involved in chromatin modifications^2^,^3^. In addition, a group of abundantly expressed lncRNAs is retained within the nucleus, forming specific nuclear bodies^4^,^5^. In general, lncRNAs exhibit a tissue-specific expression pattern, and a large fraction of them are expressed in the brain^6^. However, the physiological function of these nervous system-specific lncRNAs remains largely unknown.

Gomafu, also known as MIAT (Myocardial infarction associated transcript) and Rncr2 (retinal noncoding RNA 2), is primarily expressed in neuronal cells and localized in nuclear subdomains that do not overlap with any other subnuclear compartments^7^,^8^,^9^. A number of studies have suggested physiological roles of Gomafu in a variety of processes, including retinal cell specification and differentiation of neurons, embryonic stem cells, and excitatory neurons in embryonic brain^10^,^11^,^12^,^13^. Gomafu is also associated with a risk of myocardial infarction and schizophrenia^8^,^12^. Gomafu interacts with several RNA binding proteins including, SF1, Celf3 and QKI, and affects the splicing patterns of several genes including the schizophrenia-related genes Disc1 and Erbb4^12^,^14^,^15^. The expression of Gomafu is also downregulated in the brains of patients with schizophrenia^12^. Recent studies associate Gomafu with stress and anxiety and suggest that Gomafu can affect anxiety behaviors in mice by affecting the expression of the schizophrenia-related gene β-crystallin (Crybb1) through binding to PRC1^16^. Gomafu is also proposed to regulate transcription by acting as a competing RNA for miRNA to regulate genes associated with microvascular dysfunction^17^. However, systemic *in vivo* analysis of genetically modified mice has not been reported.

Here, we describe the creation of Gomafu knockout (KO) mice. We characterized these mice through a battery of physical and behavioral tests^18^,^19^. Although the KO mice did not exhibit obvious physical differences, they demonstrated hyperactive behaviors and enhanced responsiveness to the psychostimulant methamphetamine. The sensitivity to methamphetamine was associated with an increase in the extracellular concentration of dopamine in the nucleus accumbens of KO mice. In addition, we investigated the role of Gomafu in the gene expression pathway using RNA-seq analysis of cultured neurons. We found that Gomafu regulates a small number of genes associated with important neuronal functions, although they might not be directly related to the hyperactive phenotypes.

## Results

### Lack of anatomical abnormalities in Gomafu KO mice

**Figure 1:**
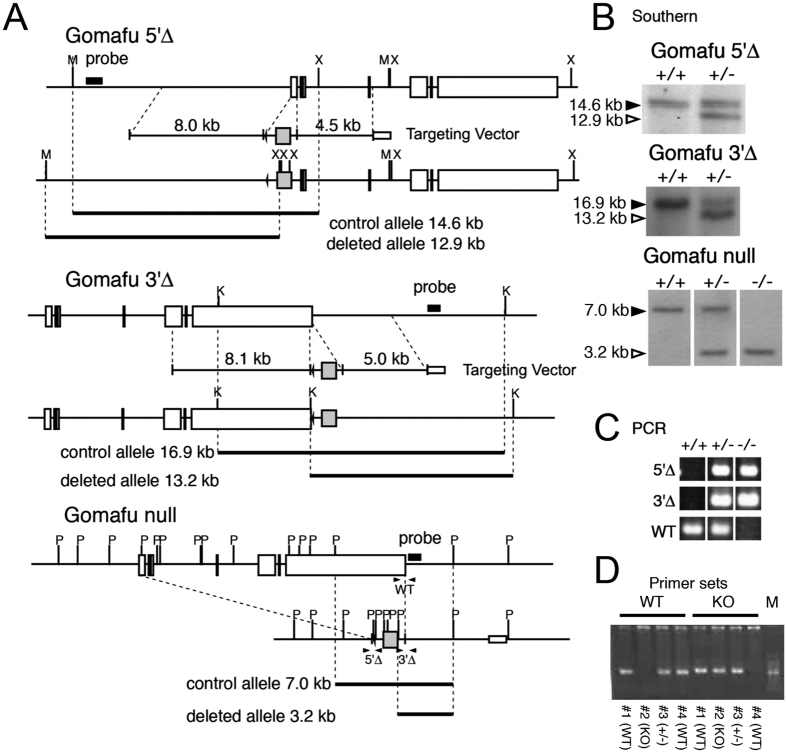
Generation of Gomafu KO mice using the TAMERE strategy. (**A**) Top and middle, schematic representations of the WT Gomafu loci and vectors that target the 5′- and 3′-exons of Gomafu and the resulting *Gomafu*^*5′∆*^ and *Gomafu*^*3′∆*^ alleles. Bottom, Schematic representation of the Gomafu null allele. (**B**) Southern blot analysis confirming the genotypes of the Gomafu 5′∆, Gomafu 3′∆ and Gomafu null mice. Positions of the probes used and the expected size of the detected bands are indicated in (**A**). (**C**) Polymerase chain reaction (PCR) analysis depicting the genotypes of the mutant mice. Locations of the primers are noted in the bottom panel of (**A**). Note that primer sets 5′Δ and 3′Δ detect the gene-targeted allele, whereas the WT primer set detects the WT allele, as shown in (**A**). (**D**) Example of PCR genotyping. The genotypes of 4 different mice (#1–4) were determined using primers that specifically detect the WT or KO allele. Marker (M) is a 100-bp ladder molecular marker. The bright band is 500 bp.

To investigate the physiological function of Gomafu, we generated mice that completely lack *Gomafu* (*Gomafu*^*null*^/+) (Fig. 1) using the trans-allelic targeted meiotic recombination (TAMERE) strategy^20^. Briefly, two gene-targeted mice harboring loxP sites in the 5′ and 3′ ends of Gomafu were generated, and the entire gene that spans 157 kb was deleted by transgenic expression of the Cre recombinase in the meiotic cells of the trans-heterozygous mice (Fig. 1).

**Figure 2:**
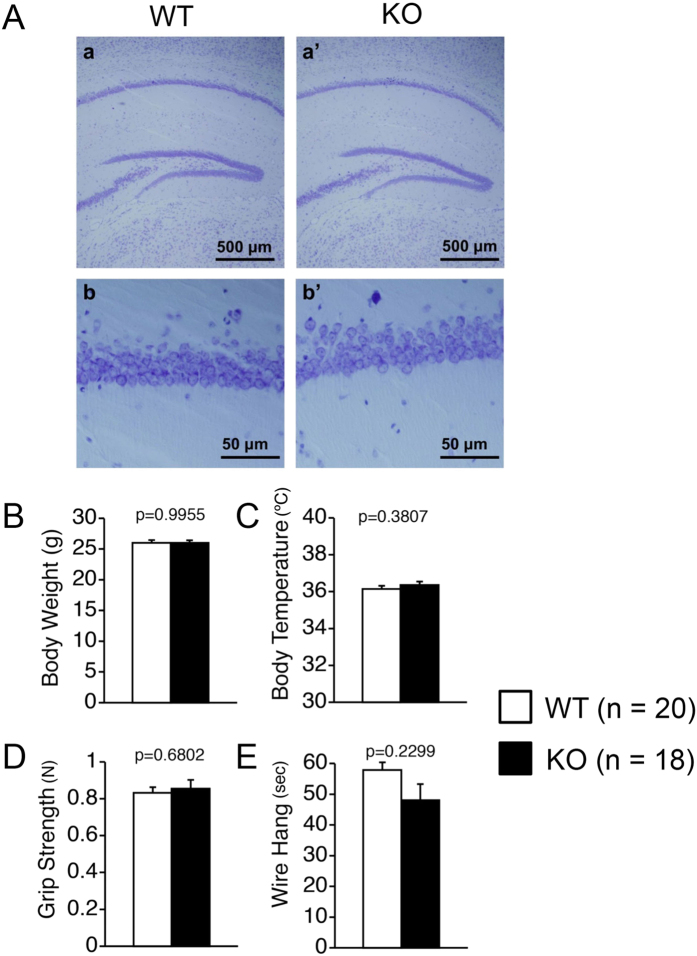
Gomafu KO mice did not show any difference from WT mice. (**A**) Nissl staining of cross sections of brains from WT and Gomafu KO mice. Hippocampi are shown in a and a’, and the CA1 regions of the hippocampi are shown in b and b’. (**B–D**) Physical characterization of WT and Gomafu KO mice. (**B**) Body weight, (**C**) Body temperature, (**D**) Grip strength and (**E**) Wire hang test. Data are represented as the means and standard errors of the mean for the indicated numbers (n) of mice, 20 WT mice and 18 KO mice. The p-value from one-way ANOVA for each test is indicated (**B–E**, genotype effect).

The homozygous *Gomafu*^*null*^/*Gomafu*^*null*^ mice (Gomafu KO mice hereafter) appeared normal and were viable and fertile. In a previous study, we found that Gomafu is highly expressed in the CA1 neurons of the hippocampus^7^. Nissl staining revealed no abnormalities in the gross anatomy of the hippocampi from the Gomafu KO mice at 3 months of age compared with wildtype (WT) of the same age (Fig. 2A(a,a’)). Moreover, no obvious morphological abnormalities were noted in the cells of the CA regions (Fig. 2A(b,b’)). This finding suggested that the lncRNA Gomafu is not required for normal mouse brain development. No significant differences in the body weight or body temperature were noted between WT and KO mice (Fig. 2B,C). Both groups also displayed similar neuromuscular strength as measured by the grip strength and wire hang tests (Fig. 2D,E). This finding suggested that the KO mice are physically similar to their WT littermates.

### Hyperlocomotion behaviors of Gomafu KO mice

Given the specific expression of Gomafu in the nervous system and the lack of obvious phenotypic defects in the Gomafu KO mice, we further assessed whether deletion of Gomafu could lead to behavioral differences. We subjected the Gomafu KO mice and their WT littermates to a comprehensive battery of behavioral test^21^,^22^.

The KO and WT mice exhibited similar performances in gait analysis, indicating that the KO mice did not have motor defects (Figure S1). We observed statistically significant changes in rotarod performance tests, although the difference was relatively small (Figure S2A). Both groups also displayed similar sensitivity to a painful stimulus as demonstrated by the hot plate test (Figure S2B). The prepulse inhibition test indicated no significant difference in sensory-motor gating between KO and WT mice (Figure S2C,D).

**Figure 3:**
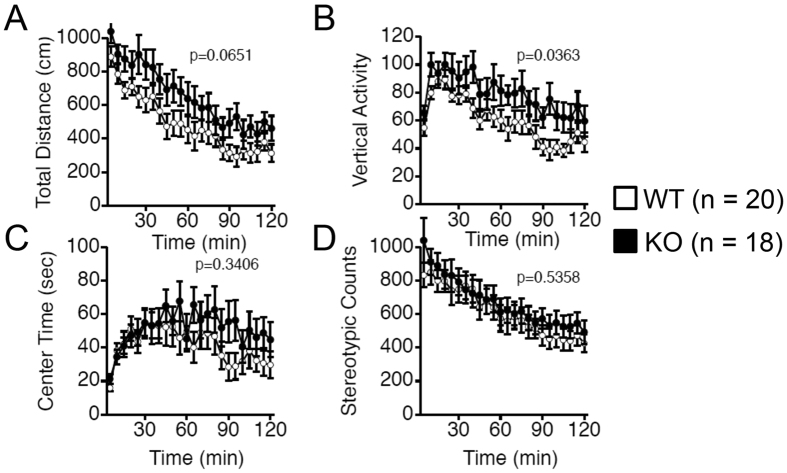
Gomafu KO mice exhibit increased locomotor activity in the open field test. (**A**) Distance traveled, (**B**) vertical activity, (**C**) time spent in the center and (**D**) stereotypic behavior counts were determined for WT and Gomafu KO mice. Data ae represented as the means and standard errors of the mean for the indicated numbers (n) of mice, 20 WT mice and 18 KO mice. The p-value from two-way repeated-measures ANOVA for each index is presented (genotype effect).

**Figure 4:**
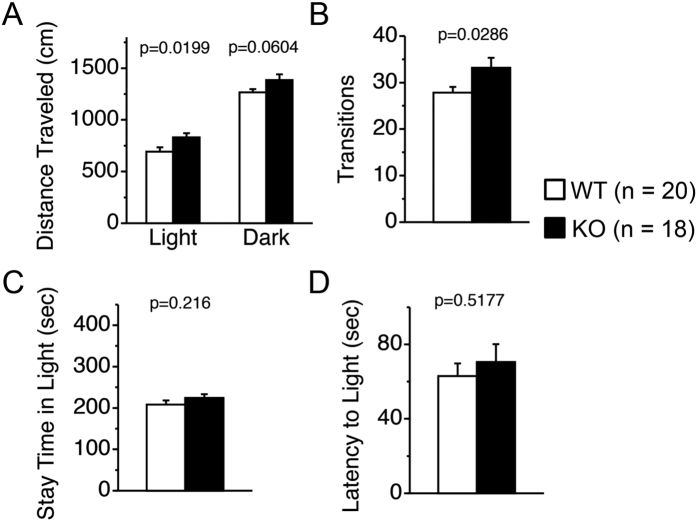
Gomafu KO mice show an increase in locomotor activity in the light-dark transition test. (**A**) Distance traveled, (**B**) transition, (**C**) time spent in light and (**D**) Latency to light were observed for a 10-minute duration. Data are represented as the means and stadard errors of the mean for the indicated numbers (n) of mice, 20 WT mice and 18 KO mice. The p-value from two-way repeated-measures ANOVA for each index is presented (genotype effect).

We next assessed the locomotive activity of the Gomafu KO and WT mice. In the open field test, Gomafu KO mice exhibited a mild increase in the distance traveled (0–30 min: p = 0.0481 from two-way repeated-measures ANOVA, F(1,34) = 4.205)) and a moderate but significant increase in vertical rearing activity compared with the WT littermates (p = 0.0363 from two-way repeated-measures ANOVA), suggesting a hyperlocomotion phenotype of the KO mice (Fig. 3A,B). However, both groups displayed similar counts of stereotypic activity, a measure of repetitive behaviors (Fig. 3D). In addition, the KO mice also exhibited a significant increase in the total distance traveled and the number of transitions in the light-dark transition test (p = 0.0099 and p = 0.0286 from two-way repeated-measures ANOVA, respectively, Fig. 4A,B). The KO mice did not exhibit a significant increase in anxiety, as indicated by the time spent at the center of an open field in the open field test as well as the time spent in the light and the latency to light in the light-dark transition test (Figs 3C and 4C,D). In the elevated plus maze test, the two groups of mice did not exhibit differences in entries into the open arms (Figure S3A,B), although the KO mice exhibited a slight increase in the time spent in the open arms (p = 0.0455 from one-way ANOVA, Figure S3D). In addition, clear depression-related behavior was not observed when the mice were subjected to a stressful environment in the Porsolt forced swim test (Figure S3E,F), although the total distance they traveled was significantly increased in KO mice at day 2 (p = 0.0588 from two-way repeated-measures ANOVA, Figure S3F). These data suggest that the hyperactive behaviors of the KO mice might not be directly related to stress or anxiety. In the social interaction test, Gomafu KO mice only showed an increase in the distance traveled without an increase in the number of social contacts, further supporting the hyperactive phenotype of the KO mice (Figure S4). Collectively, these results suggest that Gomafu deficiency caused a mild hyperlocomotive phenotype but did not affect emotional and social behaviors.

### Increased sensitivity of Gomafu KO mice to a psychostimulant

**Figure 5:**
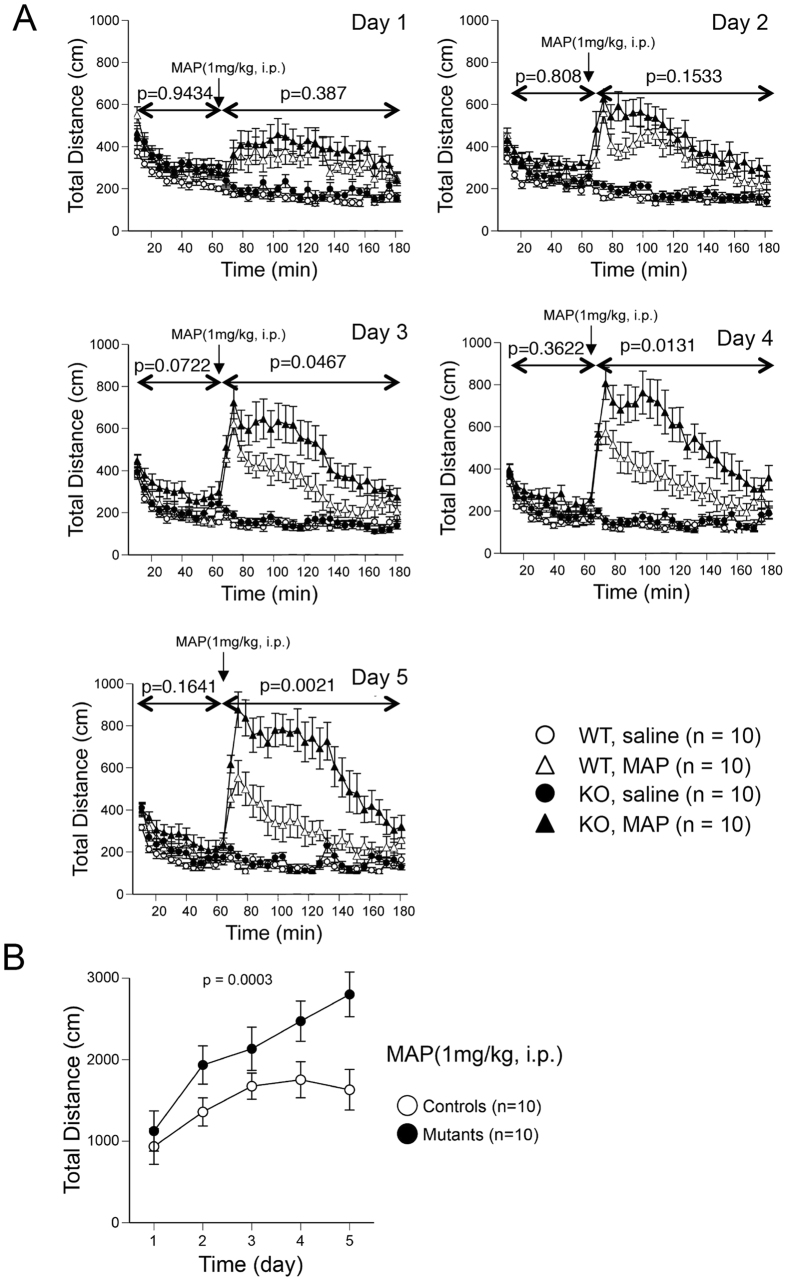
Locomotor activity of Gomafu KO mice is stimulated by methamphetamine (MAP). WT and KO mice were injected with MAP (1 mg/kg, i.p.), and their total distance traveled in open field test was recorded. (**A**) Total distance traveled by WT and KO mice injected with saline or MAP for 5 consecutive days at days 1–5. Data are represented as the means and standard errors of the mean for the indicated numbers (n) of mice, 10 for each genotype. The p-value from two-way repeated-measures ANOVA for each test (0–60 min, 60–180 min, WT with MAP vs KO with MAP) is presented. (**B**) Sensitization of locomotor response measured at 20 minutes after the administration of MAP on 5 consecutive days. The p-value from two-way repeated-measures ANOVA (WT vs KO) is presented.

Given that hyperactivity is a symptom of the psychiatric disorder schizophrenia and that Gomafu expression is downregulated in post-mortem brain samples of schizophrenia patients^12^, we were interested in the effect of psychostimulants on Gomafu KO mice. Considering that chronic methamphetamine treatment has been used as a drug-induced animal model for schizophrenia^22^,^23^,^24^,^25^, we chose to assess the effects of methamphetamine (MAP), which inhibits the dopamine transporter and thus increases the dopamine level at the synaptic clefts in the brain. Single intraperitoneal injection (i.p.) of MAP at a dose of 1 mg/kg of body weight induced increases in total distance traveled in open field test by both WT and KO mice (day 1, Fig. 5A). However, the increase in distance traveled by the KO mice did not significantly differ from the increase in their WT littermates (p = 0.387 from two-way repeated-measures ANOVA, day 1, Fig. 5A). Thus, we asked whether repeated administration of MAP affected the hyperlocomotive behaviors of WT and KO mice differently. Mice were injected with MAP at the same dose for 5 consecutive days. Both WT and KO mice exhibited progressive increases in the distance traveled from day 1 to day 5 when compared at 20 minutes after the injection of MAP (at 80 min in Fig. 5A,B, p = 0.0430 for WT and p = 0.0010 for KO from one-way repeated-measures ANOVA), which was consistent with the previous observation that repeated methamphetamine administration produces locomotor sensitization over time^25^. Notably, KO mice exhibited a greater increase than the WT mice (p = 0.0003 from two-way repeated-measures ANOVA), suggesting that Gomafu KO mice are more sensitive to the repeated MAP stimulation than WT. The genotype-dependent increase in locomotor sensitization became statistically significant after 3 days of injection (60–180 min, p = 0.0467 at day 3, p = 0.0131 at day 4, and p = 0.0021 at day 5 from two-way repeated-measures ANOVA, Fig. 5A).

**Figure 6:**
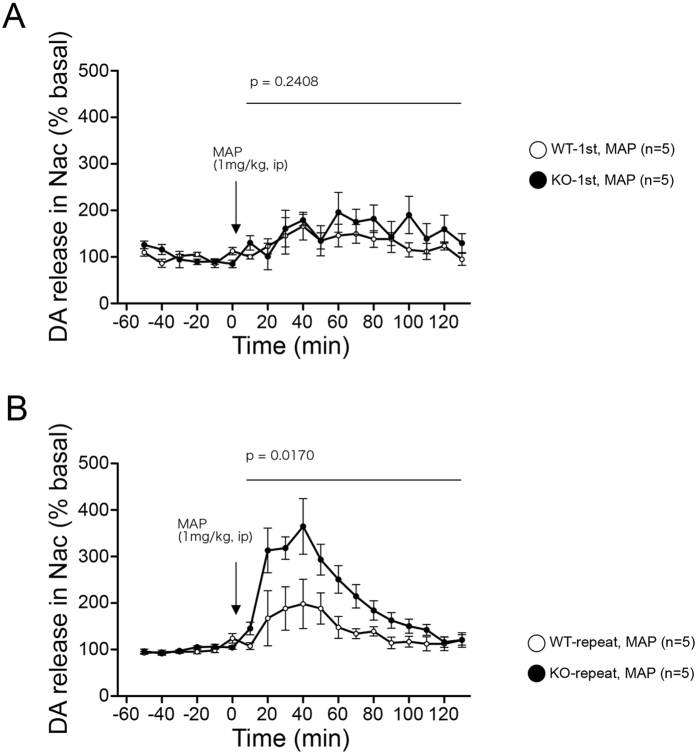
Repeated treatment with MAP induced a release of dopamine in the nucleus accumbens of Gomafu KO mice. The extracellular concentration of dopamine (DA) in the nucleus accumbens of freely moving WT or Gomafu KO mice was measured by *in vivo* microdialysis and high-performance liquid chromatography. Five basal fractions were collected before the administration of MAP. After treatment with MAP (1 mg/kg, i.p) at time 0, 6 fractions were collected every 20 minutes. Dopamine concentration was expressed as a percentage of the average of values of the 5 baseline fractions obtained. Data are represented as the means and standard errors of the mean for the indicated numbers (n) of mice, 5 for each genotype. The p-value from two-way repeated-measures ANOVA for each test (10–120 min, WT vs KO) is presented. (**A**) Dopamine concentration after a single treatment. (**B**) Dopamine concentration after repeated treatment.

Hyperactivity is typically associated with dopamine levels in the brain. Given that the Gomafu KO mice showed hyperactive behaviors and increased responsiveness to MAP, we performed *in vivo* microdialysis to determine the extracellular level of dopamine in the nucleus accumbens after administration of methamphetamine. Samples were collected from living mice with probes implanted in the nucleus accumbens, and the concentration of dopamine was determined by high-performance liquid chromatography (HPLC) before and after the mice were injected i.p. with 1 mg/kg of MAP. The increase in dopamine did not differ between WT and KO after single injections (Fig. 6A). However, after repeated injections, the dopamine level in Gomafu KO mice dramatically increased compared with levels after one injection, exhibiting statistically significant increased response compared with WT mice (p = 0.0170 from two-way repeated-measures ANOVA, Fig. 6B). On the other hand, WT mice did not exhibit such an increase in the dopamine level after repeated injection compared with that after one injection (Fig. 6B). These data suggest that Gomafu KO led to an increase in MAP-induced dopaminergic neurotransmission in the nucleus accumbens.

### Transcriptome profiling of hippocampal neurons from Gomafu KO mice

**Figure 7:**
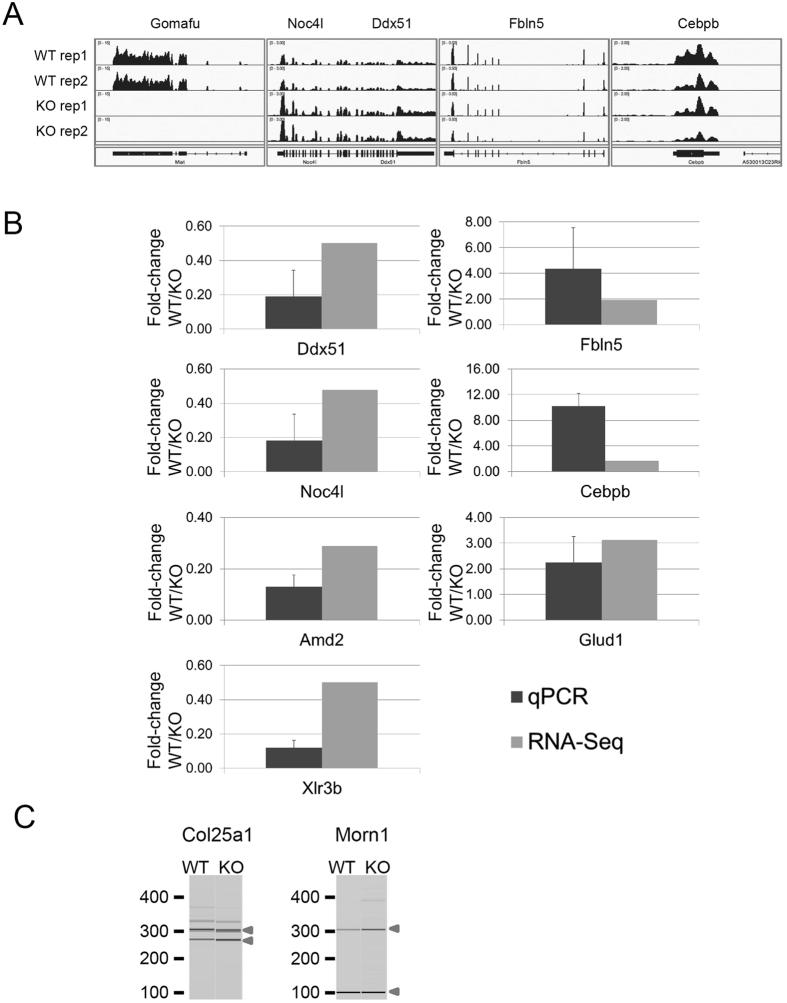
Polymerase chain reaction (PCR) analysis validating transcripts that were affect by the KO of Gomafu in neurons. (**A**) Mapping of RNAseq reads at the representative genomic loci. Note that the number of reads was increased at Noc4l and Ddx51 and decreased at Fbln5 and Cebpb. Essentially no reads were mapped to Gomafu in the KO animals. rep1 and rep2 denote replicate 1 and replicate 2, respectively. (**B**) Quantitative PCR (qPCR) validation of genes that revealed changes in their transcript levels between WT and KO neurons. For each pair of primers, the difference in transcript level was represented as the average of the fold-changes for 3 pairs of WTs and KOs (WT/KO) with a standard deviation for the qPCR and the average of the fold-changes for the two pairs that had been sequenced. Refer to Table S1 for numerical data. (**C**) Reverse transcription PCR (RT-PCR) validation of splicing changes in Gomafu KO neurons. Data are presented as gel-like images generated by Agilent Bioanalyzer. Input was RNA from a pair of WT and KO neurons that were sequenced. The included and skipped isoforms are indicated by triangles. Refer to Figure S5 and Table S2 for the results of the replicates and the numerical data.

We aimed to identify genes that caused the behavioral difference between WT and Gomafu KO mice and to study the role of Gomafu in the regulation of gene expression in neurons. Initial microarray analyses using RNA prepared from the whole brain or dissected hippocampal samples did not detect significant gene expression differences between the WT and Gomafu KO mice (Supplemental Tables T1 and T2). Given that Gomafu is expressed in a subset of neurons and not in glial cells, we speculated that the subtle changes in gene expression in the Gomafu-expressing neurons were masked by transcripts derived from the non-expressing neurons and glial cells. We therefore prepared hippocampal cultures that uniformly expressed Gomafu under serum-free conditions and in the absence of contaminating glial cells that did not express Gomafu. The Gomafu KO cultures were paired with WT cultures from the same parents. In total, we prepared 3 sets of cultures from two sets of parent mice for RNA extraction. The RNA samples from the two sets that were from the same parents were pooled together according to their genotypes to reduce the number of samples in the analysis from 6 to 4. These RNAs were then sequenced using the Illumina platform. In total, we generated approximately 250 million reads from each sample, and these reads were mapped to the mouse genome assembly mm9 (Fig. 7A).

Among all the genes that were expressed in one of the genome types (cutoff 0.58 cRPKM, 40 percentile of all the cRPKM values), 19 genes were expressed differently (1.5-fold difference) between the two genotypes (Table S3). This result indicated that the impact of knocking out Gomafu on the transcriptome was limited to a small number of genes in the context of cultured hippocampal neurons. We attempted to validate the differences in the expression of 13 known genes by quantitative polymerase chain reaction (qPCR) using samples that were sequenced plus one extra pair of samples that were siblings to one of the sequenced pairs. In total, we confirmed the difference in 7 out of 8 genes for which we were able to obtain functional and efficient primers (Fig. 7 and Table S3). Although we did not identify candidate genes that were clearly involved in the hyperactive behaviors in the KO mice, the genes affected in the Gomafu KO mice are still of interest. Among these genes, Xlr3b and Cebpb affect cognitive behavior and are involved in the proliferation of hippocampal neurons^26^,^27^. The two genes that exhibited the largest difference in expression between WT and KO were Ddx51 and Noc4l. In the murine genome, these genes are located at the same positions on different strands of chromosome 5, where Gomafu is also located. In the KO, both genes exhibited increased expression. Although both genes are approximately 1.57 Mb (megabases) away from Gomafu, it was possible that the removal of the Gomafu loci changed the local chromosomal structure, leading to changes in the expression of other genes located on the same chromosome. To assess whether Gomafu RNA rather than Gomafu DNA sequences affected Ddx51 and Noc41 expression, we established a Neuro2A (N2A) cell line that express Gomafu upon the addition of doxycycline (Dox). When Gomafu expression was induced, Ddx51 expression decreased in two independent samples, whereas Noc4l showed a slight decrease in one of the samples (Table S3). These results suggest that Gomafu transcripts negatively regulate at least Ddx51 expression, and the upregulation of this gene in Gomafu KO mice was likely not a result of changes in chromosomal structure.

Because previous studies demonstrated that Gomafu affects alternative splicing in a number of genes and reported Gomafu binding to several RNA binding proteins, we hypothesized that Gomafu is involved in the regulation of alternative splicing in neurons as previously proposed^12^,^13^. Using a custom computational pipeline that was previously used to study alternative splicing in vertebrates and recently used to discover a group of neuronal microexons that are misregulated in brains of patients with autism spectrum disorder, we aimed to identify alternative splicing events that were differently regulated between WT and KO neurons^28^,^29^. This alternative splicing pipeline covers all hypothetically possible splice junctions and is able to detect and quantify different classes of alternative splicing events, including cassette exon, alternative splice site selection and intron retention.

The output of this pipeline is a value that represents the splicing level as Percent Spliced In (PSI) and a confidence score for the PSI value. To identify splicing events that were affected by the deficiency of Gomafu, we eliminated all the events that had PSI values with a low confidence score and identified events with a difference in PSI between WT and KO that were greater than 15% in at least one of the pair and greater than 10% in the other pair. With these criteria, we identified 50 cassette type exons, 57 alternative splice sites, 33 introns and 6 complex splicing events that include more than one type of splicing changes. To validate the PSI values, 27 pairs of primers detecting different classes of AS events were designed. Using these primers, semi-quantitative reverse transcription polymerase chain reaction (RT-PCR) reactions were set up using the RNA samples that were used for high-throughput sequencing and the PCR products were visualized and quantified using a bioanalyzer. Of these 27 pairs of primers, 8 pairs produced products of expected length that were quantifiable by the bioanalyzer, suggesting that the expression level of these candidate genes were rather low. Among them, 2 pairs exhibited the same differences in the RT-PCR reaction between WT and KO, namely Col25a1 and Morn1 (Figs 7, S5 and Table S2). Although the correlation between the PSI from the pipeline and calculated from semi-quantitative RT-PCR had a correlation of 0.88, changes that were >10% could not be observed in the RT-PCR (Figure S5). This discrepancy could be caused by the differences in sensitivity between the two detection methods to detect small changes in alternative splicing. In this case, the largest difference in the predicted PSI was only ~20%, which might be too small of a difference to be detected by the less-sensitive semi-quantitative RT-PCR. Nevertheless, the majority of the splicing events in the pipeline did not exhibit significant differences between WT and KO neurons. Although Gomafu binds to various RNA proteins and splicing factors^12^,^14^,^15^, it may regulate a limited number of target genes, and alternative splicing at a global level is largely unaffected in the hippocampal neuron cultures.

## Discussion

In this study, we used Gomafu KO mice as a model to assess the physiological function of this lncRNA. Given that previous studies demonstrated that Gomafu is involved in ES cell, neuronal cell and retinal cell differentiation, it was unexpected that we did not observe any anatomical abnormality in the brains of Gomafu KO mice^12^,^13^,^30^. We also did not observe any physical difference between the KO and WT mice. However, when the mice underwent a battery of behavioral tests, they exhibited a hyperactive phenotype and increased sensitivity to the psychostimulant MAP. These two phenotypical changes were associated with increased extracellular levels of dopamine in the nucleus accumbens after injection with MAP. These data suggest that Gomafu is involved in the regulation of the mesolimbic pathway.

A recent study demonstrated that antisense-oligo-mediated gene knockdown of Gomafu in the medial prefrontal cortex in the mouse brain affected the anxiety-related behaviors of mice in the fear-conditioning test and open field test^16^. However, we could not detect any differences between the WT and KO mice in the fear-conditioning test (Figure S6), which was consistent with the observation that the KO mice did not exhibit any behavioral changes that were related to anxiety in the open field test. It is also possible that if the KO mice were assessed in the fear-conditioning test, they may display anxiety-related behaviors. This difference may be due the lack of Gomafu in a specific part of the brain, e.g., the prefrontal cortex versus the lack of Gomafu in the entire brain. We also cannot eliminate the possibility that differences in the techniques used to eliminate the expression of Gomafu caused this discrepancy. Nevertheless, both murine models with deficiencies in Gomafu exhibited increases in the distance traveled in the open field test, indicating that the lack of Gomafu led to a hyperlocomotive phenotype in mice.

Interestingly, this hyperactive behavior of the Gomafu KO mice was enhanced when the mice were treated with the MAP. It remains unclear how the KO of Gomafu led to increase in sensitivity to MAP. Our data also indicate that the extracellular concentration of dopamine increased in the nucleus accumbens of Gomafu KO mice after treatment with MAP, suggesting that the enhanced activity involves the increase in dopamine transmission. Consistent with this idea, several KO mice models exhibit a hyperactive phenotype that is enhanced by psychostimulants and is associated with an increase in the extracellular concentration of dopamine in the nucleus accumbens and striatum^22^,^31^. However, the mechanism by which Gomafu affects dopamine transmission remains to be elucidated. Several studies have demonstrated changes in Gomafu expression after stimulations by psychostimulants in both mouse and human. Gomafu is downregulated in the nucleus accumbens of mice treated with MAP^32^. However, Gomafu is upregulated in the nucleus accumbens of cocaine and heroin users^33^,^34^. Nevertheless, Gomafu may be involved in the molecular pathway that is responsible for substance abuse. Gomafu has been implicated in the psychiatric disease schizophrenia. Gomafu is downregulated in the cortex of schizophrenia patients, and the knockdown of Gomafu in neurons can affect the splicing patterns of schizophrenia-related genes DISC1 and ERBB4^12^. Hyperactivity is a symptom of the psychiatric disease schizophrenia^35^. In addition, patients with schizophrenia are more sensitivity to psychostimulants, including MAP^36^. The behavioral changes in the Gomafu KO mice further support the relationship between Gomafu and schizophrenia.

To identify genes that are regulated by Gomafu and responsible for the behavioral changes, we performed RNA-seq on RNA collected from primary hippocampal neuron cultures of WT and Gomafu KO mice. In general, the lack of Gomafu did not affect the transcript levels or the alternative splicing patterns of most genes in the mouse genome. In total, we identified 19 genes with expression changes and 146 alternative splicing changes. Although we did not identify candidate genes that could be directly associated with the behavioral changes in the KO mice, some of the affected genes are involved in important neuronal functions. Among the genes with expression changes that were validated by qRT-PCR, Cebpb (CCAAT-enhancer binding protein β) is a transcription factor that is involved in consolidation of memory in hippocampus through an autoregulatory feedback loop by Bdnf^37^. Cebpb is also involved in the regulation of neuronal apoptosis, thus allowing for the proliferation of neurons^27^,^38^. Xlr3b (X-linked lymphocyte-regulated 3B) is an imprinting gene that affects cognitive behaviors in mice^26^.

Although we did not address the mechanism by which Gomafu affect these genes, two recent studies provide some evidence. Gomafu interacts with major components of PRC1 (polycomb repressive complex 1) and BMI1 to control the expression of a neighboring gene Crybb1 in response to fear conditioning, leading to changes in mouse behavior^16^. This finding suggests that Gomafu could also regulate Ddx51 and Noc4l, two genes that are located on the same chromosome of Gomafu, through this type of regulation. Another study demonstrated that Gomafu acts as a competing RNA (ceRNA) to control the expression of mRNA that share the same miRNA binding sites as Gomafu^17^. In this study, the authors demonstrated that Gomafu controls the expression of VEGF mRNA during angiogenesis by competing for miR150-5p, thus leading to increased VEGF expression^17^. Further bioinformatics studies are necessary to determine whether the genes affected by Gomafu are targets of miRNAs that are able to bind to Gomafu. In addition, we cannot eliminate the possibility that some of the identified genes are not directly regulated by Gomafu or that the changes in their expression are secondary effects caused by changes in other genes.

Gomafu affects the splicing of a handful of genes, including the schizophrenia-related genes DISC1 and ERBB4 as well as the neurogenesis-related gene Wnt7b^12^,^13^. Therefore, we also attempted to identify splicing changes in the RNA-seq data that were caused by knocking-out Gomafu. Among the genes with splicing changes in the Gomafu KO cells, we did not identify the genes that were previously reported to be regulated by Gomafu. This discrepancy can be caused by the differences between the neurons with Gomafu expression manipulated transiently and the neurons collected from hippocampi of Gomafu KO mice. Nevertheless, we identified 2 splicing events that are persistently affected by Gomafu deficiency. In the Gomafu KOs, exon 29 of Col25a1 (CLAC-P/collagen XXV), which encodes part of a collagen triple helix repeat, exhibited more skipping (Table S2, Figs 7 and S5). Col25a1 is a transmembrane-type collagen that regulates intramuscular motor innervation, and the COL25A1 gene is associated with antisocial personality disorder and substance dependence^39^,^40^. However, further studies are required to determine whether Gomafu directly regulates the splicing of Col25a1 and whether this change in splicing is responsible for the changes in the behavior of the KO mice. In addition, Gomafu affected the selection of a 5′ splice site in Morn1 (MORN repeat containing 1), a gene with unknown function in mammals. In the neurons of the KO mice, an alternative splice site is used more often, leading to inclusion of an extra 206 nucleotides, thus disrupting the open reading frame of the mRNA.

Given that Gomafu binds to several splicing factors^12^,^14^,^15^, it is surprising that knocking out Gomafu only affects a small fraction of splicing events in the mouse transcriptome. This finding may be because different regions in the brain involved in the reward circuitry are connected in a complex manner and cultured primary neurons alone cannot recapitulate the splicing changes that are responsible for the different behaviors between WTs and KOs. Alternatively, Gomafu may affect restricted gene loci located in close proximity to the Gomafu complex. It would thus be intriguing to investigate genome-binding sites of Gomafu using recently reported methods^41^,^42^,^43^, which might clarify the molecular mechanism of Gomafu in gene regulation.

## Methods

All the experiments were performed in accordance with the safety guidelines of RIKEN.

### Generation of Gomafu KO mice

Gomafu KO mice were generated using TAMERE technology^20^. First, we generated embryonic stem (ES) cells with either the first exon (*Gomafu*^*5′∆*^) or the polyadenylation signal (*Gomafu*^*3′∆*^) in one of the Gomafu alleles were replaced by a loxP-PGK-Neo cassette using previously described protocols (http://www.cdb.riken.jp/arg/Methods.html). Chimeric mice were generated with the recombinant ES clones and mated with C57BL/6 females to generate heterozygous animals (*Gomafu*^*5′∆*^*/*+ (Acc. No. CDB0438K) and *Gomafu*^*3′∆*^*/*+ (Acc. No. CDB0439K)). These mice were mated with transgenic mice that expressed the Cre recombinase under the control of Sycp1 (Synaptonemal Complex protein 1) promoter (B6;D2-Tg(Sycp1-cre)4 Min/J, Jackson laboratory) to generate double heterozygous mice, which were subsequently inter-crossed to generate *Gomafu* trans-heterozygous (*Gomafu*^*5′∆*^/*Gomafu*^*3′∆*^) mice with the Sycp1-Cre transgene. Due to the expression of the Cre recombinase, these males produced sperm that have the alleles completely deleted (*Gomafu*^*null*^), and we obtained a single *Gomafu*^*null*^/+ mouse. This mouse was extensively backcrossed to C57BL/6 to match the genetic background, and the congenic background was confirmed with primers that detect single nucleotide polymorphisms (SNPs) in C57BL/6, CBA, and DBA2. The congenic mice were further backcrossed to C57BL/6 to obtain animals used for the behavior assay. The genotypes of the KO mice were confirmed by both Southern blot analysis and PCR analysis of genomic DNA. We used the following primers for genotyping: WT FW, 5′- cttgtttgagcaggacactgtgcgag-3′, WT RV: 5′- ccaatcttggctcaccagcaactc-3′, KO FW, 5′-gcctctccactggccagcgt-3′, KO RV, 5′-gcggtgctgtccatctgcacgagac-3′. The following PCR conditions were employed: pre-denature at 96 °C for 1 minute followed by 35 cycles of denaturation at 94 °C 30 for sec, annealing at 62 °C for 30 sec, and elongation at 72 °C for 30 sec. The animals were housed in cages and were provided with unlimited food and water and a 12-hour light and 12-hour dark cycle. All behavioral tests were performed with male mice that were at least seven weeks old when the tests began. All the animal protocols were approved by the safety division of RIKEN.

### Histological staining

Whole brains were dissected from WT and Gomafu KO mice at the age of 8 weeks and were snap-frozen with optimal cutting temperature (OCT) compound (Sakura Finetek, Torrance, CA) before 20-μm-thick cross-sections were made with a Cryostat (Thermo Scientific). The sections were then fixed with 4% paraformaldehyde and stained with 0.1% Cresyl violet solution.

### Behavioral testing

Behavioral testing was performed as previously described^21^,^22^,^44^. The data are presented as the means and standard errors of the means and were analyzed by analysis of variance (ANOVA) or two-way repeated-measures ANOVA using StatView software (SAS Institute, Cary, NC). Raw data from the behavioral tests, the date on which each experiment was performed, and the age of the mice at the time of the experiment are available in the Mouse Phenotype Database (http://www.mouse-phenotype.org/).

### General health & neural sensitivity test

For a general health test, the body weight and body temperature of the mice were recorded. The grip strength test and wire hang test were used to assess neuromuscular strength. In the grip strength test, the forelimb grip strength was assessed by a grip strength meter (O’Hara & Co., Tokyo, Japan), which recorded the peak force applied by the forelimbs of a mouse in Newtons (N). Briefly, a mouse was held by the tail with its forepaws grasping a vertical wire grid. Then, mice were pulled back gently by the tail with their body parallel to the surface of the table until the wire grid was released. Each mouse was tested thrice, and the largest value recorded was used for analysis. In the wire hang test, a mouse was placed on a wire mesh and was allowed to grip the wire. Then, the wire mesh was inverted and waved gently and the latency to fall was recorded after 60 sec.

### Open field test

The open field test was used to measure the locomotor activity of the mice. A mouse was placed at the center of the open field apparatus (40 × 40 × 30 cm, Accuscan Instruments, Columbus, OH), and data were collected for 120 minutes. We recorded total distance traveled (in cm), vertical activity (rearing movement measured by counting the number of photobeam interruptions), time spent at the center of the apparatus and stereotyped activity (counts of beam breaks).

### Light dark transition test

The apparatus for the light-dark transition test was a cage (21 × 42 × 25 cm) that was divided into two equal chambers by a partition with a door (O’Hara & Co.). One side was brightly illuminated (390 lux), whereas the other was dark (2 lux). A mouse was placed in the dark chamber and was allowed to move freely between the two sides through the door for 10 minutes. The total number of transitions, time spent on each side, the distance traveled and the latency to enter the light chamber for the first time were recorded by ImageLD4 software (O’Hara & Co.)^45^.

### MAP administration and microdialytic quantitation of dopamine

Microdialysis was performed as described previously^22^,^46^. Briefly, the animals were anesthetized with sodium pentobarbital, and surgery was performed to implant a guide cannula (AG-4; EICOM, Kyoto, Japan) into the nucleus accumbens. On the following day, a dialysis probe (AI-4-1, 1-mm membrane length; EICOM) was inserted through the guide cannula and was perfused with artificial cerebrospinal fluid (148 mM NaCl, 2.7 mM KCl, 1.2 mM CaCl2, and 0.85 mM MgCl2) at a flow rate of 1 μl min–1. After an equilibration period of one hour, samples were collected every 20 minutes. After collection of at least five baseline fractions, mice were administered MAP (1 mg kg–1, i.p.), and 6 more fractions of samples were collected. The concentration of dopamine in the samples was measured by HPLC on a 4.6 by 30 mm PP-ODS column (EICOM) that was maintained at 25 °C and equipped with an electrochemical detection system (HTEC-500, EICOM) and PowerChrom (EICOM).

### Mouse primary neurons and cell cultures

Primary hippocampal neurons were cultured as described previously^47^. In brief, hippocampi were dissected from E16.5 mouse embryos and treated with 0.25% trypsin in Ca2+- and Mg2+-free saline buffered with Hepes (HCMF, pH7.4) for 10 min at 37 °C. After two washes with HCMF, trypsinized tissues were dissociated into single cells by mild pipetting, and cells from a pair of hippocampi were plated into 4 poly-lysine coated dishes (3.5 cm) (Thermo Fisher Scientific). The cells were maintained in Neurobasal medium supplemented with B27 supplement and glutamine. Half of the medium was changed every three days. Cells were harvest for RNA extraction with Trizol (Life Technologies) after 10 days *in vitro*. To establish Neuro2A cells that conditionally express Gomafu, Neuro2A cells were co-transfected with pT2K-rtTA-M2, pT2K-TRE-Gomafu-pA, and pCAG-T2TP^48^, and single colonies were assessed for Gomafu expression. The cells were maintained in Eagle’s Minimum Essential Medium supplemented with 10% fetal bovine serum and penicillin-streptomycin. Gomafu expression was induced with 500 ng/ml doxycycline for 24 hours before harvesting for RNA collection.

### RNA sequencing and analysis

RNA sequencing and analysis were performed as described elsewhere^28^,^29^. Polyadenylated RNA was extracted from two pairs of WT and Gomafu KO neurons and was used to create the sequencing libraries. The libraries were sequenced using the Illumina Genome Analyzer II following the protocol of manufacturer, and sequencing reads were extracted using the standard GA pipeline software v1.4 (Illumina). The reads were mapped to the mouse genome (mm9) using Bowtie. The expression level for each gene was determined as a corrected version of reads per kilobase of target transcript sequence per million of total reads (cRPKM). For splicing analysis, a customized analysis pipeline was used that could align reads to a library of exon-exon junctions and then quantify alternative splicing events^28^,^29^,^49^. Splicing level for each alternative splicing event is represented as a PSI (Percent Spliced In).

### Quantitative PCR and RT-PCR

For quantitative PCR (qPCR), 2 μg of total RNA was used for cDNA synthesis with a ReverTra Ace® qPCR RT Kit (Toyobo). Briefly, 2 to 7.5 μl of the 200 μl of resultant cDNA was used as input in qPCR using THUNDERBIRD SYBR qPCR Mix (Toyobo). The reactions were run with a melting temperature of 58 °C for 40 cycles in the ABI 7900HT Fast Real-Time PCR System (Applied Biosystems), and the data were analyzed using the absolute quantification method. For RT-PCR, 5 to 20 ng of total RNA was used as input and reverse transcription and amplification was performed using the One-Step RT-PCR Kit (Qiagen) according to manufacturer’s protocol. The reactions were run with a melting temperature of 58 °C for 28 cycles. The reactions were then analyzed by a 2100 Bioanalyzer (Agilent) with the Agilent DNA 1000 Kit.

## Additional Information

**How to cite this article**: Ip, J. Y. *et al.* Gomafu lncRNA knockout mice exhibit mild hyperactivity with enhanced responsiveness to the psychostimulant methamphetamine. *Sci. Rep.* **6**, 27204; doi: 10.1038/srep27204 (2016).

## Supplementary Material

Supplementary Information

Supplementary table T1

Supplementary table T2

Supplementary table T3

Supplementary table T4
