# Supplementary material for: Gomafu lncRNA knockout mice exhibit mild hyperactivity with enhanced responsiveness to the psychostimulant methamphetamine
Source: Sci Rep. 2016 Jun 2;6:27204. doi: 10.1038/srep27204 (PMC4890022; doi:10.1038/srep27204)
Supplement: Supplementary Information [file srep27204-s1.pdf]

Supplementary Information for
Gomafu lncRNA knockout mice exhibit mild hyperactivity with enhanced responsiveness to the psychostimulant methamphetamine

Joanna Y Ip, Masamitsu Sone, Qun Pan, Kiyoyuki Kitaichi, Kaori Yanaka, Takaya Abe, Keizo Takao, Tsuyoshi Miyakawa, Benjamin J. Blencowe and Shinichi Nakagawa

Supplementary Methods

Behavioural Testing

Behavioural testing was performed as described in (Miyakawa, Leiter et al. 2003, Morishima, Miyakawa et al. 2005, Sakae, Yamasaki et al. 2008). The data are presented as means and standard errors of the means and were analyzed by two-tailed Student's t-test, Scheffe's test, analysis of variance (ANOVA) or two-way repeated-measures ANOVA using StatView software (SAS Institute, Cary, NC).

Gait analysis test

In the gait analysis, the DigiGait™ Imaging System (Mouse Specifics Inc, Watertown, MA), was used to analysis the gait of mice during spontaneous walk/trot locomotion. Using this system, the motions of mice on a transparent motorized treadmill were recorded on video and the accompanying software automatically identified the components of the stride and calculated the width of the stance, the length of the stride, the step angle and the paw angle.

Rotarod test

A mouse was places on a rotating drum (3 cm in diameter) and the time that the mouse was able to maintain its balance on the rod was measured. The rotarod (Accelerating Rotarod, UGO Basile, Collegeville, PA) was accelerated from 4 to 40 rpm over a period of five minutes.

Elevated plus maze test

The elevated plus-maze was a plus-shaped apparatus consisted of two oppositely located open arms and two oppositely located enclosed arms and a central square. The maze was made of white plastic plates and was elevated 55 cm above the floor. The sides of the open arms are surrounded by 3-mm-helight Plexiglass walls to prevent the mice from falling off the apparatus. A mouse was placed in the central square, facing one of the enclosed arms and its behaviour was recorded for 10 mins. The percentage of entries into open arms, the time spent in open arms (s), the total number of arm entries and the total distance traveled (cm) were recorded and determined. Collection and analysis of data were performed using ImageEP software (O'Hara & Co.) (Komada et al., 2008).

Porsolt forced swimming test

The apparatus is made up of four plastic cylinders that are 20 cm in height and 10 cm in diameter. The cylinders were filled with water at 23°C up to a height of 7.5 cm. Mice were placed into the cylinders, and their behavior was recorded over a 10-min test period. ImagePS software was used for data acquisition and analysis.

Social interaction test

Two mice of the same genotypes that had not been house together were placed in a box (40 by 40 by 30 cm) and allowed to explore freely for 10 min and their behavior was monitored by a CCD camera. ImageSI software was used to perform automatic analysis. An active contact is considered as when the mice contacted each other and either one of them traveled at least 5 cm. Parameters that were measured included total duration of contacts, number of contacts, number of active contacts, mean duration per contact, and total distance traveled.

Startle response prepulse inhibition test

The startle response and prepulse inhibition test were performed using a startle reflex measurement system (O'Hara & Co.). At the beginning of the test, a mouse was placed in a plastic cylinder and was left undisturbed for 10 minutes. For all trial types, white noise (40 ms) was used as the startle stimulus. Starting with the onset of the prepulse stimulus, the startle response was recorded for 140 ms (with measurement of the response every 1 ms. The background noise level in each chamber was 70 dB and the dependent variable was the peak startle amplitude recorded during the 140-ms sampling window.

Each test session had six trial types - two types of startle stimulus only trial and four types of prepulse inhibition trial. The prepulse sound was presented at 74 or 78dB. Hundred ms after the prepulse sound, startle stimulus with intensity at 110 or 120 dB was presented. The following four combinations of prepulse and startle stimuli were used - 74 and 110, 78 and 110, 74 and 120, and 78 and 120 dB. Six blocks of the six trial types were presented in pseudorandom order to ensure that each trial type was presented once within a block. The interval between trials ranges from 10 to 20 s with an average of 15 s.

Supplementary Figure Legends

Figure S1. Gomafu knockout (KO) mice did not show any significant difference from the wildtype (WT) mice in the Gait analysis

(A) Motion of the front paw. (B) Motion of the hind paw. Data are represented as means and standard errors of the mean for the indicated numbers (n) of mice - 20 wildtype mice and 18 knockout mice. P-value from one-way ANOVA is presented.

Figure S2. Gomafu knockout (KO) mice did not show any deficit in motor coordination and sensorimotor gating

(A) Latency to fall from the rotarod test. P-value from one-way ANOVA is presented. (B) Latency measured by the hot plate test. P-value from two-way repeated measures ANOVA is presented. (C, D) Amplitude of the startle response (C) and (D) percentage of prepulse inhibition

from startle response prepulse inhibition test. Data are represented as means and standard errors of the mean for the indicated numbers (n) of mice - 20 wildtype (WT) mice and 18 knockout mice. P-value from two-way repeated measures ANOVA is presented.

Figure S3. Gomafu knockout (KO) mice did not show any significant increase in anxiety or depressive behaviour

In the elevated plus maze test, (A) number of entries, (B) percentage of entries into open arms, (C) distance traveled and (D) percentage of time on open arms were recorded. (E) Percentage of immobility and (F) distance traveled in the Porsolt forced swim test. Data are represented as means and standard errors of the mean for the indicated numbers (n) of mice - 20 wildtype mice and 18 knockout mice. P-value from one-way ANOVA (A-D) and two-way repeated measures ANOVA (E, F) is presented.

Figure S4. Gomafu knockout (KO) mice did not show any abnormal social behaviour in the social interaction test

(A) Total duration of contact, (B) number of contacts, (C) total duration of active contacts, (D) mean duration per contact and (E) distance traveled. Data are represented as means and standard errors of the mean for the indicated numbers (n) of mice - 20 wildtype mice and 18 KO mice. P-value from one-way ANOVA is presented.

Figure S5. Reverse transcription PCR (RT-PCR) validation of splicing changes in Gomafu knockout (KO) neurons

(A) Data is shown as gel-like images generated by Agilent Bioanalyzer. Input was RNA from the two pairs of wildtype and knockout neurons that had been sequenced. The included and skipped isoforms were indicated by triangles. (B) Scatter plot showing Present Splice In (PSI) (%) values from RT-PCR versus RNA-Seq for the two pairs of wildtype and knockout neurons for all the alternative splicing events that were we were able to design functional primers.

Figure S6. Gomafu konkcout (KO) mice did not show anxiety related behavior in the fear-conditioning test

Levels of freezing time during conditioning, context testing, and cued testing with altered context in the Gomafu WT (Controls) and KO (Mutants) mice. No significant difference was observed. P-value for each test is shown as p.

Supplementary Reference

Komada, M., Takao, K., Miyakawa, T. Elevated Plus Maze for Mice. J. Vis. Exp. (22), e1088, doi:10.3791/1088 (2008).

Miyakawa, T., L. M. Leiter, D. J. Gerber, R. R. Gainetdinov, T. D. Sotnikova, H. Zeng, M. G. Caron and S. Tonegawa (2003). "Conditional calcineurin knockout mice exhibit multiple abnormal behaviors related to schizophrenia." Proc Natl Acad Sci U S A **100**(15): 8987-8992.

Morishima, Y., T. Miyakawa, T. Furuyashiki, Y. Tanaka, H. Mizuma and S. Nakanishi (2005). "Enhanced cocaine responsiveness and impaired motor coordination in metabotropic glutamate receptor subtype 2 knockout mice." Proc Natl Acad Sci U S A **102**(11): 4170-4175.
Sakae, N., N. Yamasaki, K. Kitaichi, T. Fukuda, M. Yamada, H. Yoshikawa, T. Hiranita, Y. Tatsumi, J. Kira, T. Yamamoto, T. Miyakawa and K. I. Nakayama (2008). "Mice lacking the schizophrenia-associated protein FEZ1 manifest hyperactivity and enhanced responsiveness to psychostimulants." Hum Mol Genet **17**(20): 3191-3203.

Supplementary table T1
Microarray analyses of gene expression changes in the whole brain of Gomafu WT and KO mice

Supplementary table T2
Microarray analyses of gene expression changes in the dissected hippocampi of Gomafu WT and KO mice

Supplementary table T3
Gene list that exhibited expression changes in the Gomafu KO neurons and qPCR validation

Supplementary table T4
Gene list that exhibited alternative splicing changes in the Gomafu KO neurons and qPCR validation

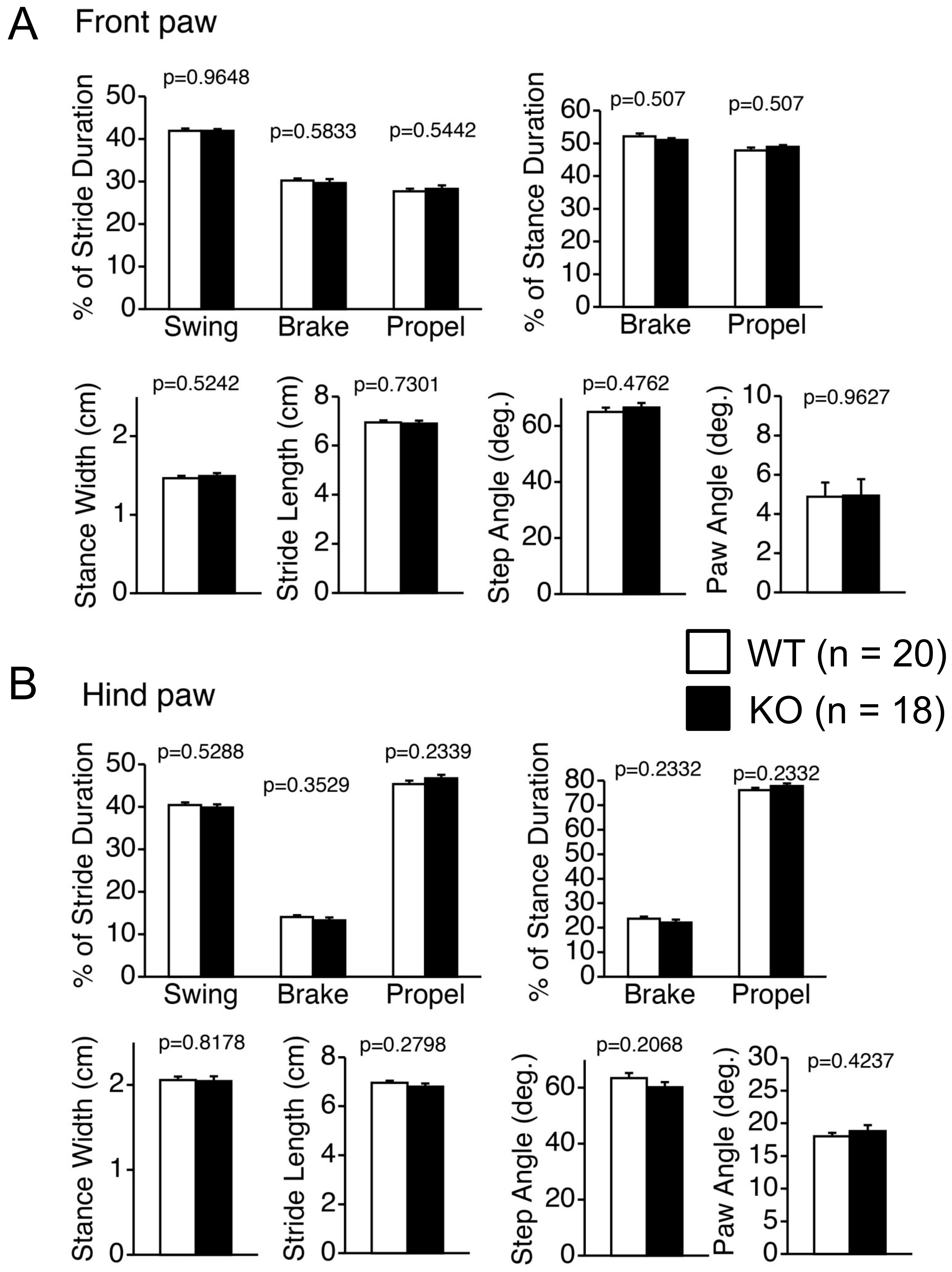
A
Front paw
p=0.9648
p=0.5833
p=0.5442
% of Stride Duration
Swing
Brake
Propel
p=0.507
p=0.507
% of Stance Duration
Brake
Propel
p=0.5242
Stance Width (cm)
p=0.7301
Stride Length (cm)
p=0.4762
Step Angle (deg.)
p=0.9627
Paw Angle (deg.)
WT (n = 20)
KO (n = 18)
B
Hind paw
p=0.5288
p=0.3529
p=0.2339
% of Stride Duration
Swing
Brake
Propel
p=0.2332
p=0.2332
% of Stance Duration
Brake
Propel
p=0.8178
Stance Width (cm)
p=0.2798
Stride Length (cm)
p=0.2068
Step Angle (deg.)
p=0.4237
Paw Angle (deg.)

Figure S1

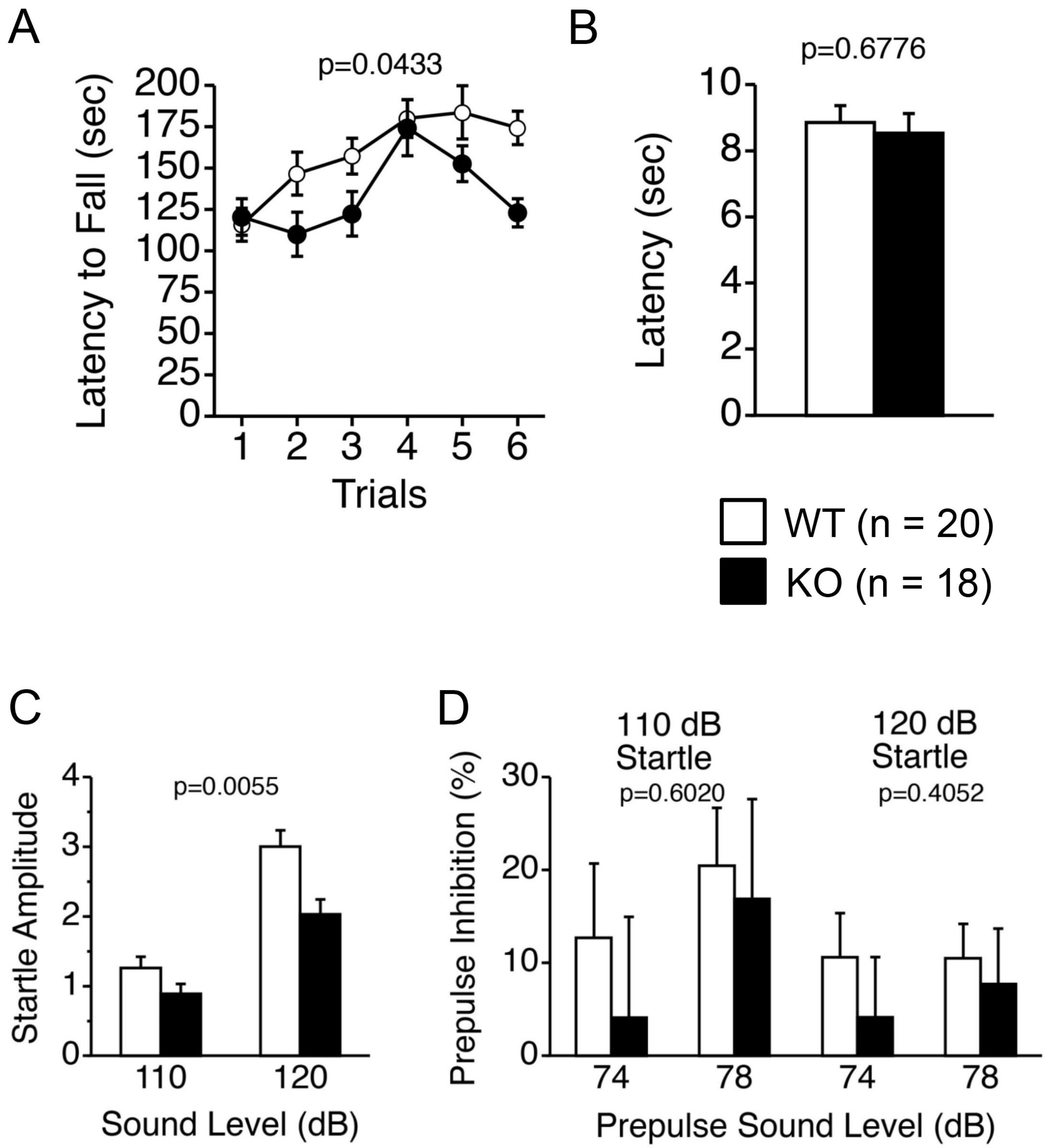

A
p=0.0433
Latency to Fall (sec)
200
175
150
125
100
75
50
25
0
1
2
3
4
5
6
Trials
B
p=0.6776
Latency (sec)
10
8
6
4
2
0
WT (n = 20)
KO (n = 18)
C
p=0.0055
Startle Amplitude
4
3
2
1
0
110
120
Sound Level (dB)
D
110 dB Startle
p=0.6020
120 dB Startle
p=0.4052
Prepulse Inhibition (%)
30
20
10
0
74
78
74
78
Prepulse Sound Level (dB)

Figure S2

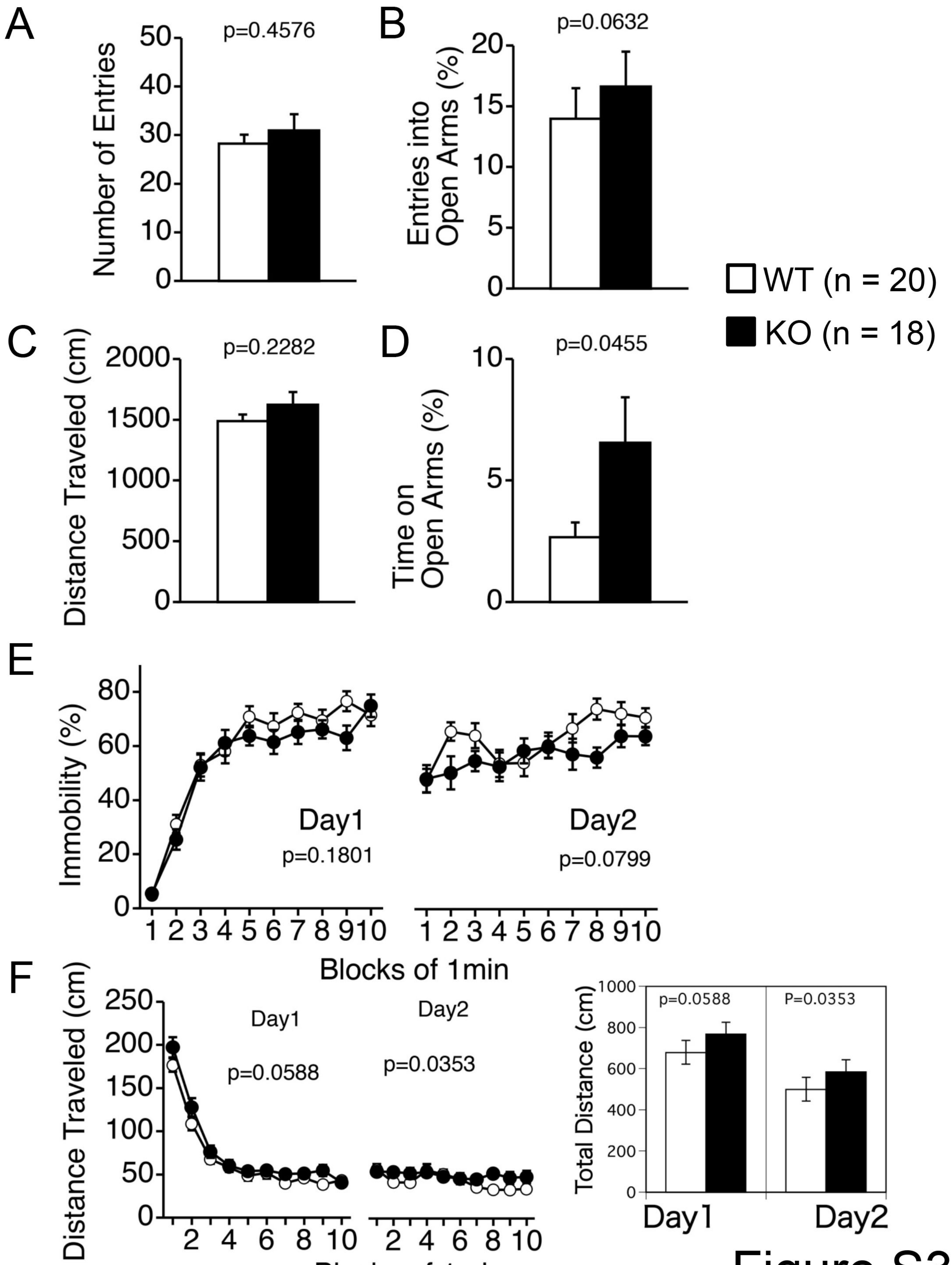
A
p=0.4576
Number of Entries
B
p=0.0632
Entries into Open Arms (%)
WT (n = 20)
KO (n = 18)
C
p=0.2282
Distance Traveled (cm)
D
p=0.0455
Time on Open Arms (%)
E
Immobility (%)
Day1
p=0.1801
Day2
p=0.0799
Blocks of 1min
F
Distance Traveled (cm)
Day1
p=0.0588
Day2
p=0.0353
Blocks of 1min
Total Distance (cm)
p=0.0588
P=0.0353
Day1
Day2

Figure S3

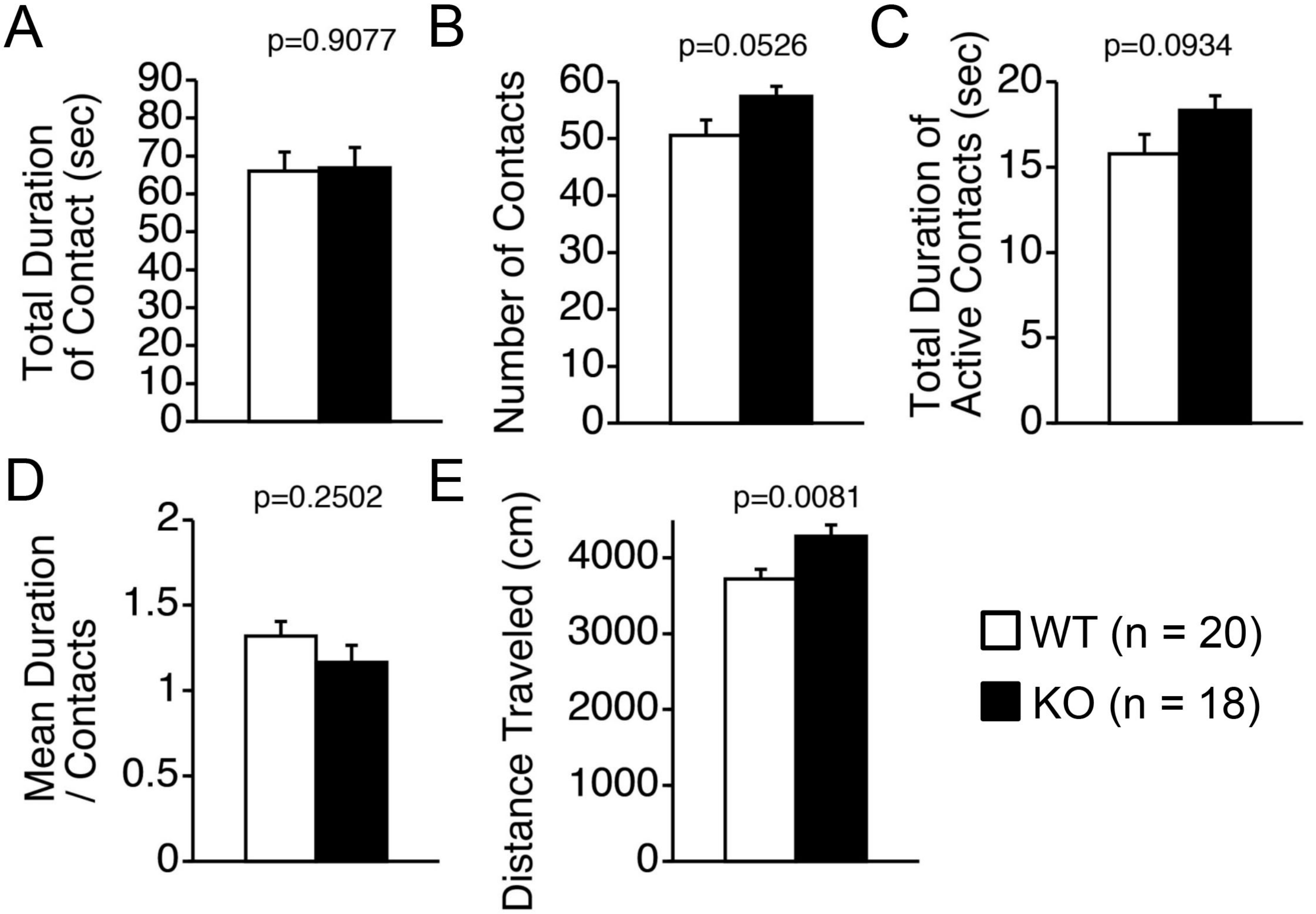
A
p=0.9077
Total Duration of Contact (sec)
90
80
70
60
50
40
30
20
10
0
B
p=0.0526
Number of Contacts
60
50
40
30
20
10
0
C
p=0.0934
Total Duration of Active Contacts (sec)
20
15
10
5
0
D
p=0.2502
Mean Duration / Contacts
2
1.5
1
0.5
0
E
p=0.0081
Distance Traveled (cm)
4000
3000
2000
1000
0
WT (n = 20)
KO (n = 18)

Figure S4

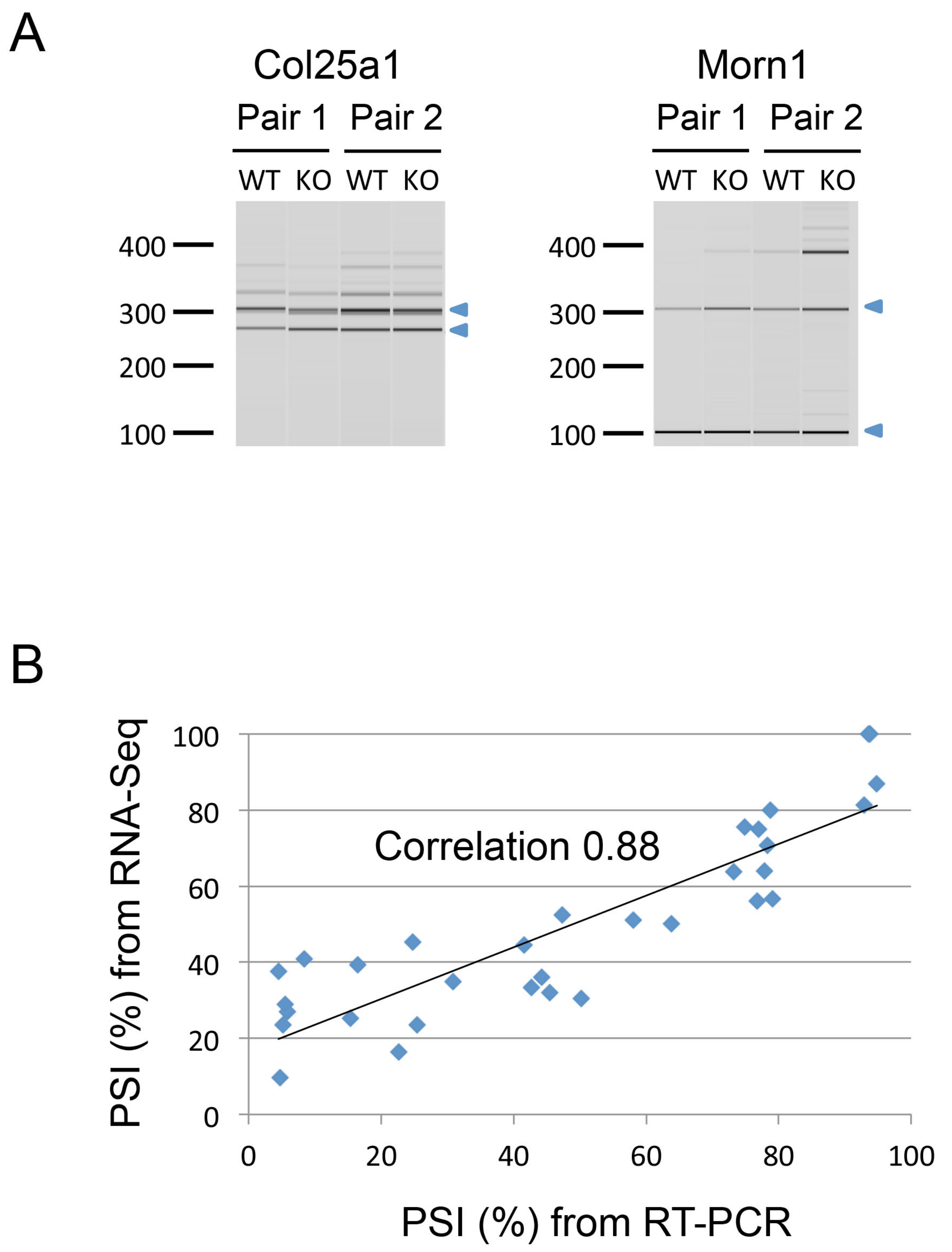
A
Col25a1
Pair 1
Pair 2
WT KO WT KO
400
300
200
100
Morn1
Pair 1
Pair 2
WT KO WT KO
400
300
200
100
B
PSI (%) from RNA-Seq
PSI (%) from RT-PCR
Correlation 0.88
0
20
40
60
80
100

Figure S5

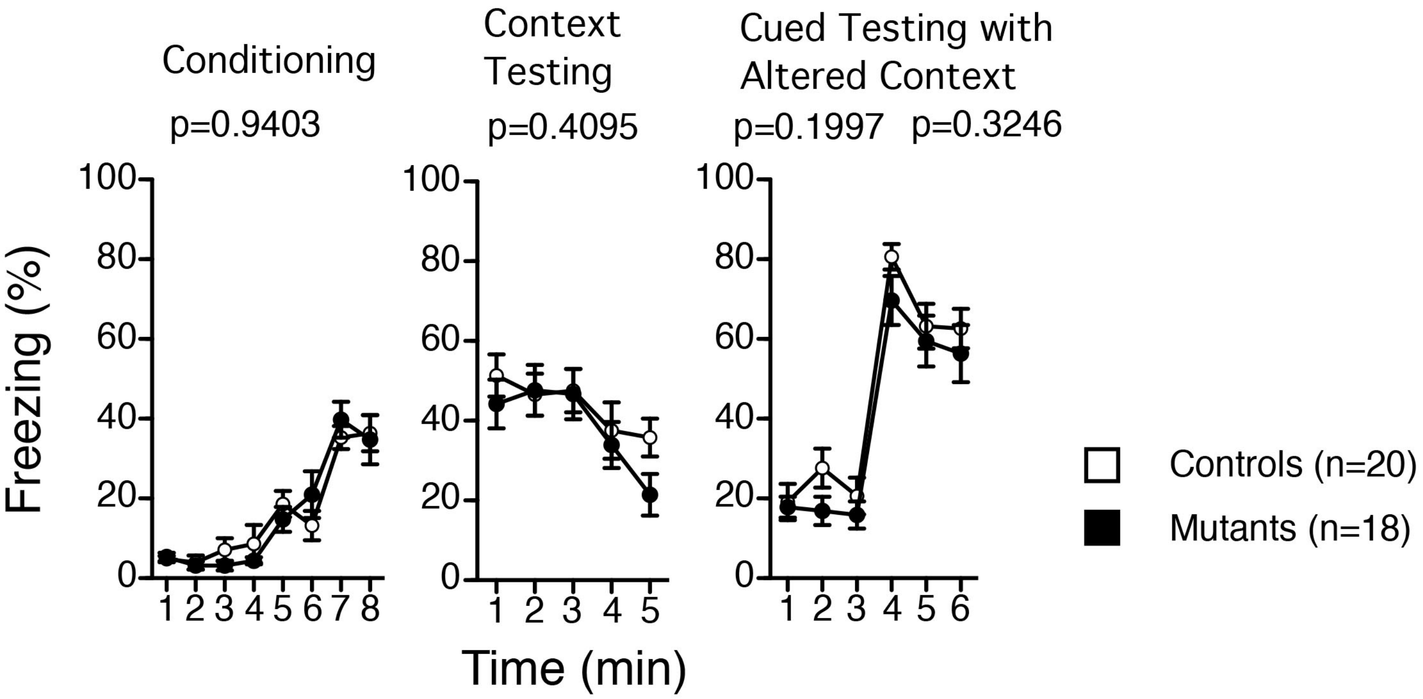
Conditioning
p=0.9403
Context
Testing
p=0.4095
Cued Testing with
Altered Context
p=0.1997
p=0.3246
Freezing (%)
100
80
60
40
20
0
1 2 3 4 5 6 7 8
1 2 3 4 5
1 2 3 4 5 6
Time (min)
Controls (n=20)
Mutants (n=18)

Figure S6
